# The Immunosignature of Mother/Fetus Couples in Gestational Diabetes Mellitus: Role of HLA-G 14 bp ins/del and PAPP-A A/C Polymorphisms in the Uterine Inflammatory Milieu

**DOI:** 10.1155/2017/4254750

**Published:** 2017-06-01

**Authors:** Miryam Martinetti, Fausta Beneventi, Cristina Capittini, Elena Locatelli, Margherita Simonetta, Chiara Cavagnoli, Irene De Maggio, Annalisa De Silvestri, Annamaria Pasi, Arsenio Spinillo

**Affiliations:** ^1^Immunogenetics Laboratory, Immunohematology and Transfusion Center, IRCCS Policlinico San Matteo Foundation, Pavia, Italy; ^**2**^ Department of Obstetrics and Gynecology, IRCCS Policlinico San Matteo Foundation and University of Pavia, Pavia, Italy; ^3^Clinical Epidemiology and Biometric Unit, IRCCS Policlinico San Matteo Foundation, Pavia, Italy

## Abstract

We enrolled 151 healthy mother/newborn couples and 26 with gestational diabetes mellitus (GDM). HLA-G and PAPP-A plasma levels were measured by ELISA at first and second trimesters, at delivery, and in cord blood. HLA-G 14 bp ins/del and PAPP-A A/C polymorphisms were genotyped. HLA-G del/del and PAPP-A C/C genotypes were more frequent among GDM mothers than controls. We observed a genetic epistasis between the two polymorphisms: the HLA-G del/del and PAPP-A C/C combination was carried by 8% of GDM mothers and 1.3% of controls (OR = 9.5, 95% CI = 0.8–109, *p* = 0.07). GDM mothers showed increased sHLA-G levels compared to controls (*p* = 0.004), and those carrying the HLA-G del/del genotype produced more sHLA-G at the second trimester and at delivery (*p* = 0.014). A genetic pressure by fetal genotype on maternal sHLA-G production was observed in GDM mothers with heterozygous HLA-G del/ins newborns (*p* = 0.02). Babies born to GDM mothers showed higher sHLA-G concentrations compared to those born to healthy mothers, and those carrying HLA-G del/del showed the highest sHLA-G levels (*p* = 0.013). PAPP-A amounts significantly increased along pregnancy (*p* < 0.001), but the median levels at the first and second trimesters were significantly lower in GDM (*p* = 0.03). Our findings first suggest an involvement of HLA-G and PAPP-A gene-protein interaction in GDM and highlight a possible contribution of the fetus in balancing maternal inflammation.

## 1. Introduction

Gestational diabetes mellitus (GDM) is a pregnancy-related complication defined as glucose intolerance associated to maternal decreased insulin sensitivity and increased insulin resistance [1]. Even though the pathophysiologic mechanisms underlying GDM are still unknown, the placenta in GDM women shows abnormal structural changes causing immature villous structure and hypervascularization and disturbances in intervillous circulation. If not properly diagnosed and treated, GDM can determine the occurrence of various complications, both in the mother and fetus [2].

In early pregnancy, two proteins are important in promoting decidual vascularization: the nonclassical human leukocyte antigen- (HLA-) G class I molecule and the pregnancy-associated plasma protein A (PAPP-A). HLA-G can stimulate the production of angiogenic factors and cytokines that favor embryo implantation, placental vascularization, and maternal-fetal tolerance [3]. PAPP-A is a metalloproteinase secreted by syncytiotrophoblast with proteolytic activity against insulin-like growth factor-binding protein-4, an important regulator of insulin growth factor bioavailability that plays a central role in fetal development and maternal well-being [4].

Due to their role as key regulators in placentation and subsequent pregnancy outcome, low blood concentrations of soluble HLA-G (sHLA-G) and PAPP-A have been associated with increased risks of miscarriage, preeclampsia, hypertension, and intrauterine growth restriction [5–10]. Besides, PAPP-A and sHLA-G maternal blood concentrations have been associated with GDM development. In particular, we previously demonstrated the direct relationship between PAPP-A and glucose metabolism in a case-control study of women at the first trimester of pregnancy, revealing a significant correlation between low levels of PAPP-A and GDM onset [11]. Furthermore, we verified that sHLA-G concentrations were higher and PAPP-A concentrations lower in GDM pregnancies than in controls, identifying a role for soluble HLA-G as an inflammation marker in pregnancies complicated by GDM [12]. Both case-control and cohort studies have found that abnormal PAPP-A and sHLA-G maternal blood levels are predictive markers of GDM [11–13].

The HLA-G gene (6p21.3) shows a polymorphic site in the 3′ untranslated region (UTR) characterized by an insertion or deletion of 14 bp (rs66554220) which affects mRNA stability and consequently the expression of the HLA-G protein [14]. The HLA-G 14 bp ins/ins genotype has been associated to increased susceptibility to spontaneous abortion, unsuccessful in vitro fertilization, preeclampsia, recurrent miscarriage, and autoimmune diseases [15–19]. The PAPP-A gene (9q33.1) shows a missense A/C single-nucleotide polymorphism (rs7020782) located on the exon 14 and generates an amino-acidic change from Ser to Tyr in the control protein module-1, possibly affecting the cell proteolytic activity of PAPP-A to the cell surface. The PAPP-A A/C polymorphism has been associated to an increased risk of recurrent pregnancy loss [20].

It is widely accepted that GDM is related to both genetic and environmental factors [1]. Therefore, this pilot study aims at verifying if the HLA-G 14 bp insertion/deletion and the PAPP-A A/C polymorphisms are involved in genetic predisposition to GDM and, above all, if a genetic epistasis between them increases the GDM risk. The results are also discussed in correlation with protein levels in both mothers and newborns looking for possible gene-disease interactions.

## 2. Materials and Methods

### 2.1. Study Population and Design

This is a cohort study that lasted from June 2011 to June 2015 and includes 177 healthy singleton pregnancies enrolled during the first trimester at the Department of Obstetrics and Gynecology of the IRCCS Policlinico San Matteo Foundation in Pavia (Italy).

We enrolled consecutively 151 healthy women and 26 who developed GDM during the observation period. The 75 g 2 h glucose tolerance test (OGTT) performed at 24–28 weeks of pregnancy was used for gestational diabetes mellitus (GDM) diagnosis. Patients were treated either by dietary adjustment or by insulin therapy. All mothers signed written informed consent for voluntary participation, and the study was approved by the Medical Ethical Committee of the IRCCS Policlinico San Matteo Foundation in Pavia. All subjects were Caucasians.

### 2.2. Molecular Analysis of HLA-G and PAPP-A Polymorphisms

One hundred and seventy-five mothers (151 healthy and 24 with GDM) and 155 newborns (135 born to healthy mothers and 20 to GDM mothers) were genotyped for the HLA-G and PAPP-A polymorphisms. DNA was extracted from the whole peripheral blood of pregnant women and from umbilical the cord blood of newborns by an automated DNA extraction system (ARROW DNA, Frysjaveien go, N-0884 Oslo, Norway). We analyzed the 14 bp insertion/deletion (ins/del) polymorphism located in the 3′ untranslated region (3′UTR) of the HLA-G gene by polymerase chain reaction (PCR), as described elsewhere [21]. We analyzed the PAPP-A A/C (Ser/Tyr) single-nucleotide polymorphism (rs7020782) located in exon 14 by real-time polymerase chain reaction (RT-PCR) using the Roche LightCycler® 480 instrument, as described elsewhere [20].

### 2.3. Soluble HLA-G Blood Plasma Concentrations

Soluble HLA-G (sHLA-G) levels were successfully measured in the maternal blood plasmas from the first to the second trimesters and at delivery and in the cord blood plasmas of the corresponding newborns. The levels of both soluble HLA-G5 and HLA-G1 molecules were detected by ELISA, following the manufacturer's instructions (Exbio/BioVendor, Praha, Czech Republic).

### 2.4. PAPP-A Blood Serum Concentrations

PAPP-A protein is synthesized by the trophoblast, and it is routinely dosed during the first trimester of pregnancy to assess the risk of Down syndrome or other fetal aneuploidies. PAPP-A levels were measured in the maternal blood sera collected at the same time as sHLA-G using the Delfia Xpress analytical platforms, following the manufacturer's instructions (PerkinElmer, Waltham, MA, USA). As PAPP-A is absent in newborns, the dosage was not performed in cord blood.

### 2.5. Statistical Analysis

The Shapiro-Wilk test was used to test the normal distribution of quantitative variables; thus, quantitative variables were expressed as median and interquartile range (IQR). Qualitative variables were summarized as counts and percentages. For quantitative variables, the differences between the two groups were evaluated by means of *t*-test for independent data or Mann–Whitney *U* test, while chi square or Fisher's exact test was used for qualitative ones. The comparisons of different genotypes between GDM and controls were performed with logistic regression, and the results were reported as odds ratio (OR) with their 95% confidence interval (CI).

A correspondence analysis was performed to represent the relationships among neonatal and maternal clinical and genetic factors and DGM onset depicting them by points in a multidimensional space, thus providing a graphic interpretation of the most important patterns underlying the data set: the nearer the dots the more strengthened the correlations. This method identifies a bidimensional space in which the data structure is represented in a simple but not discordant way with respect to the real multidimensional structure of the data distribution [22]. The particular algorithm used for calculating the degree of data dispersion can define and analyze the total variability, or inertia, of the data into a small number of dimensions or axes. The primary axis represents the significant trends in the data set, while the secondary axis shows chance variations. Plotting the axes in a two-by-two manner yields bidimensional figures. Each axis is formed by the combination of the categories, or points, that have been subjected to analysis. The reliability of the deductions depends on the amount of variability, or inertia, that can be accounted for by each axis. Thus, the percentage variability explained by each axis is reported, and the points or characteristics that show the beat concordant with this determination are indicated.

The association among sHLA-G and PAPP-A (at I and II trimesters and delivery) concentrations, different HLA-G and PAPP-A genotypes, and groups (GDM or control) was done with quantile linear regression for repeated measures, inserting also interaction terms between trimesters and diagnosis or genotype and diagnosis to study different temporal trends of protein concentrations or different behaviors of genotype in GDM mothers and healthy mothers.

The correlation between cord blood sHLA-G and maternal sHLA-G concentrations at delivery were evaluated by Spearman coefficient.

The Hardy-Weinberg equilibrium (HWE) was calculated by using the Markov chain method with exact *p* value estimation.

All tests are two sided. Data analysis was performed with STATA statistical package (release 13, 2013, Stata Corporation, College Station, Texas, USA).

## 3. Results

### 3.1. Clinical Data

We enrolled 151 voluntary healthy pregnant women at first trimester and 26 with gestational diabetes mellitus (GDM) diagnosed at 24–28 weeks of gestation. The incidence of GDM in our population is 5-6% of annual pregnancies. The main clinical characteristics of GDM pregnant women and healthy pregnant women (controls) are reported in Table 1. GDM mothers were significantly overweight (body mass index (BMI) ≥ 25) with respect to controls (*p* = 0.005), and 6 (27%) delivered babies with a birth weight > 90 centiles (adjusted for gestational age, sex, and birth order) (*p* = 0.01). Gestational age was significantly lower in GDM mothers than in controls (*p* = 0.01) (Table 1).

### 3.2. HLA-G 14 bp Insertion/Deletion and PAPP-A A/C Polymorphisms

The frequencies of HLA-G 14 bp insertion/deletion alleles and genotypes are reported in Table 2. Frequencies were in Hardy-Weinberg equilibrium. No significant deviations have been found in GDM mothers and corresponding babies versus controls (healthy mothers and babies born to healthy mothers). Only a slight increase of the HLA-G 14 bp del/del genotype frequency was found in both GDM mother (OR = 1.7) and babies born to GDM mothers (OR = 2.1), although without statistical significance.

The distribution of PAPP-A A/C alleles and genotypes is also reported in Table 2. Frequencies were in Hardy-Weinberg equilibrium. The PAPP-A C/C genotype was more frequent in GDM mothers than in healthy mothers (OR = 2.5) and was more frequent in babies born to GDM mothers than in babies born to healthy mothers (OR = 1.8), although without statistical significance.

### 3.3. Epistatic Interaction between HLA-G 14 bp Insertion/Deletion and PAPP-A A/C Polymorphisms

We verified the possible genetic epistasis between HLA-G 14 bp ins/del and PAPP-A A/C genotypic combinations on the proneness to GDM (Table 3). The frequency of carriers of HLA-G del/del and PAPP-A C/C genotypes was higher in GDM pregnant women (OR = 9.5) and in their babies (OR = 2.8) compared to the corresponding controls (healthy mothers and babies born to healthy mothers), although without reaching statistical significance.

### 3.4. Multivariate Analysis

**Figure 1:**
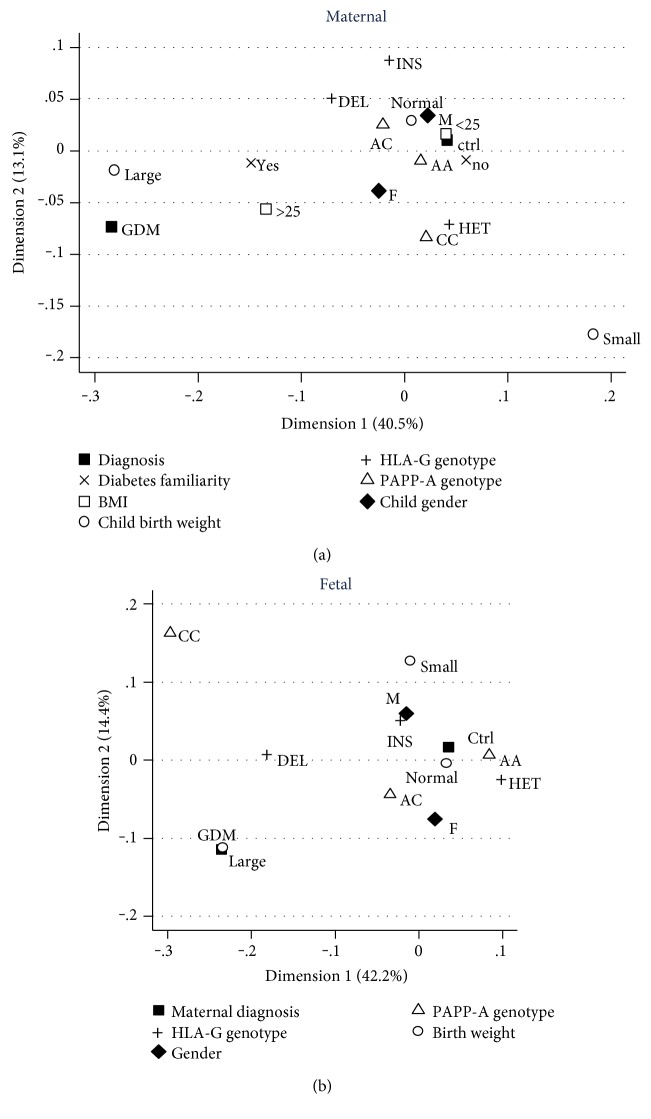
Multivariate analysis in mothers and babies using the multiple correspondence analysis (MCA) plot. (a) The variables related to mothers; (b) those related to babies.

We plotted the clinical and genetic data into a bidimensional chart using the multiple correspondence analysis (MCA) (Figure 1). At the top, there are depicted data from the mothers, and at the bottom, the data from the babies.

The variables related to GDM mothers were having BMI > 25, familiarity for diabetes and babies over 90th centile. The main covariates, distinguishing children born to GDM mothers from those born to healthy mothers, were being large for gestational age and carrying HLA-G del/del and PAPP-A C/C genotypes.

### 3.5. Maternal sHLA-G Plasma Levels and Correlation with Maternal and Neonatal HLA-G 14 bp Insertion/Deletion Genotypes

**Figure 2:**
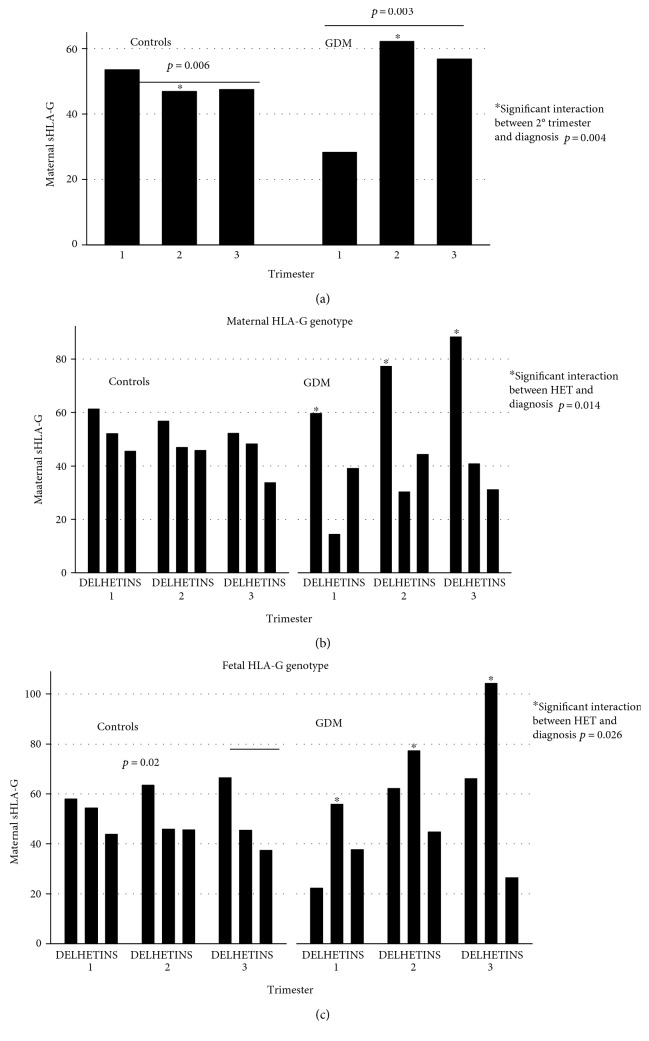
Maternal soluble HLA-G concentrations (ng/mL) in the first and second trimesters of pregnancy and at delivery in controls and GDM (a). Correlation with maternal HLA-G 14 bp insertion/deletion (ins/del) genotype in the first and second trimesters of pregnancy and at delivery in healthy mothers (controls) and GDM mothers (b). Correlation with fetal HLA-G 14 bp insertion/deletion (ins/del) genotype in the first and second trimesters of pregnancy and at delivery in healthy mothers (controls) and GDM mothers (c).

Soluble HLA-G (sHLA-G) plasma concentrations were measured in GDM and healthy mothers at the first and second trimesters of pregnancy and at delivery (Figure 2(a)). During the trimesters, the median concentration of sHLA-G increased in GDM mothers (*p* = 0.003), while decreased in controls (*p* = 0.006). The highest median sHLA-G concentration was found in the second trimester of GDM (significant interaction between 2° trimester and diagnosis; *p* = 0.004*β*55, 95% CI = 17–93) (Figure 2(a)).

Considering the gene-disease interactions, we observed that all the mothers with HLA-G del/del genotype were the highest producers of sHLA-G independently from the trimesters. In particular, a statistically significant increased production of sHLA-G was observed in all the trimesters in GDM patients carrying the HLA-G del/del genotype (significant interaction between DEL and diagnosis; *p* = 0.014*β*39, 95% CI = 1–78) (Figure 2(b)).

Cross-correlating the maternal plasma levels with the fetal HLA-G genotype, we noticed a peak in GDM women with an HLA-G del/ins fetus, while in controls, the peak was in pregnant women with an HLA-G del/del fetus (significant interaction between HET and diagnosis; *p* = 0.026*β*35.6, 95% CI = 4–66), particularly evident in the second trimester (*p* = 0.02) (Figure 2(c)).

### 3.6. Cord Blood sHLA-G Levels and Correlation with Fetal and Maternal HLA-G 14 bp Insertion/Deletion Genotypes

**Figure 3:**
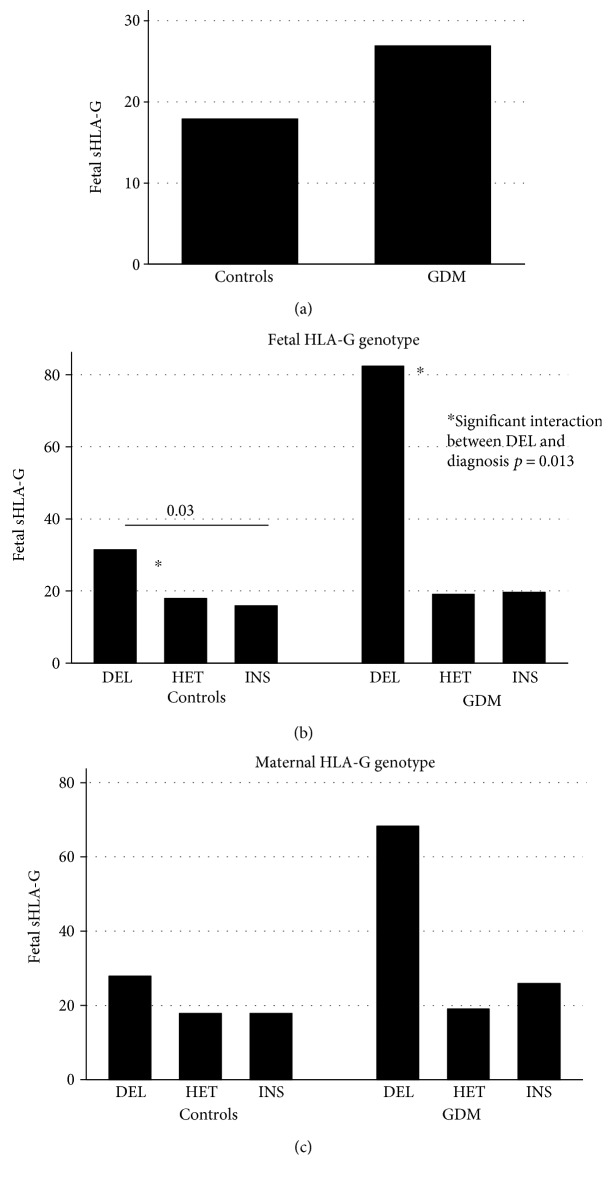
Fetal soluble HLA-G concentrations (ng/mL) in babies born to GDM and healthy mothers (a). Correlation with fetal HLA-G 14 bp insertion/deletion (ins/del) genotype in babies born to GDM and healthy mothers (b). Correlation with maternal HLA-G 14 bp insertion/deletion (ins/del) genotype in GDM and healthy mothers (c).

Soluble HLA-G plasma concentrations were measured in neonates born to GDM mothers and to controls. We found higher sHLA-G concentrations in newborns born to GDM women compared to those born to healthy mothers (median value 26.9 ng/mL versus 17.9, *p* = 0.25) (Figure 3(a)).

Then, we verified the correlation between the HLA-G 14 bp ins/del polymorphism and sHLA-G levels in cord blood plasma from babies born to GDM and healthy mothers. The highest sHLA-G levels were observed in babies carrying the HLA-G 14 bp del/del genotype, either born to GDM or to controls (*p* = 0.03). Considering the gene-disease interactions, a statistically significant peak of sHLA-G was detected in the cords of HLA-G del/del newborns of diabetic mothers (significant interaction between DEL and diagnosis; *p* = 0.013*β*29, 95% CI = 6–52) (Figure 3(b)).

Cross-correlating the cord blood plasma levels with the HLA-G maternal genotype, the highest sHLA-G levels were in babies with an HLA-G del/del mother (*p* = 0.25*β*12.9, 95% CI = −9 + 35) (Figure 3(c)).

### 3.7. Correlation between Maternal PAPP-A Serum Levels with Maternal and Neonatal PAPP-A A/C Genotypes

**Figure 4:**
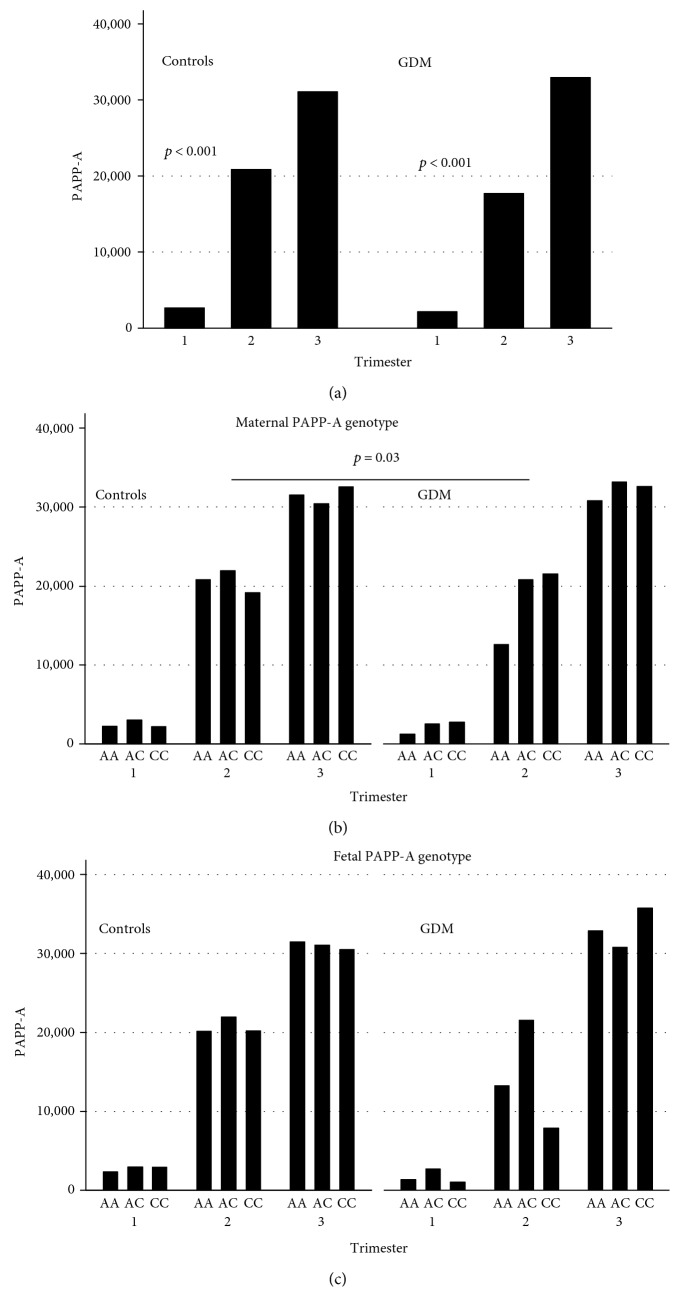
Maternal PAPP-A serum concentrations (mU/L) in the first and second trimesters of pregnancy and at delivery in controls and GDM (a). Correlation with maternal PAPP-A A/C genotypes in the first and second trimesters of pregnancy and at delivery in controls and GDM (b). Correlation with fetal PAPP-A A/C genotypes in the first and second trimesters of pregnancy and at delivery in controls and GDM (c).

The amounts of maternal PAPP-A (PAPP-A is not measurable in cord blood) significantly increased along the pregnancy as in controls and in GDM (second versus first trimester, *p* < 0.001; at delivery versus first trimester, *p* < 0.001) with a similar ascending trend in the two settings (*p* = 0.67) (Figure 4(a)). However, the median levels at the first and second trimesters were lower in GDM than in healthy pregnancies.

We correlated the PAPP-A serum levels with the PAPP-A A/C maternal genotypes. A statistically significant difference was noticed in GDM with respect to controls, in particular during the second trimester (*p* = 0.03) (Figure 4(b)).

Finally, we correlated the maternal PAPP-A serum levels with the neonatal PAPP-A genotypes. We did not find any trends that might suggest an influence of neonatal PAPP-A A/C polymorphism on maternal PAPP-A production (*p* = 0.66) (Figure 4(c)).

### 3.8. Correlation between Neonatal and Maternal sHLA-G Concentrations at Delivery in GDM and Healthy Pregnancies

**Figure 5:**
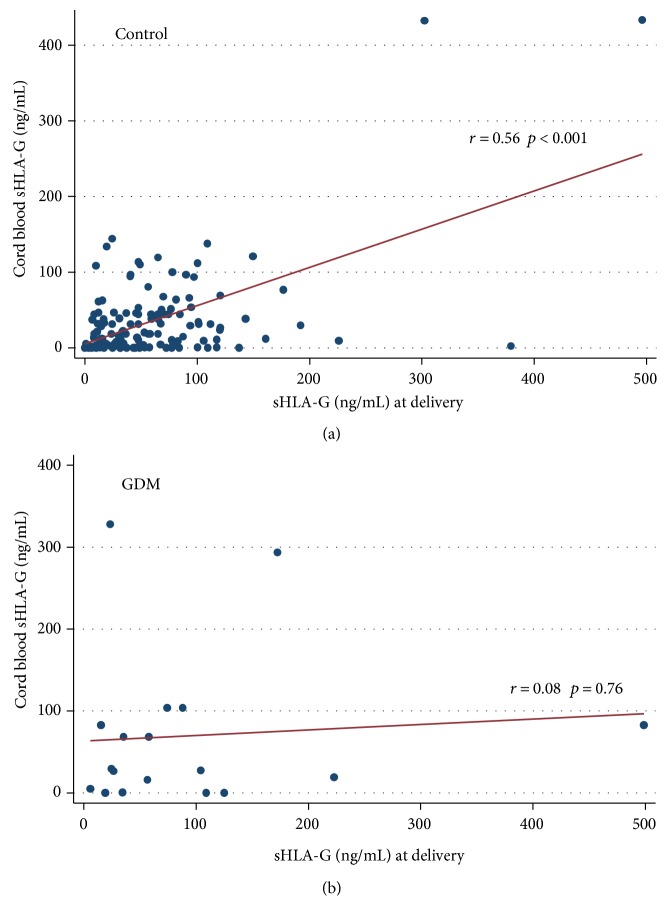
(a) Correlation indexes of sHLA-G (ng/mL) between mother at delivery and cord in controls. (b) Correlation indexes of sHLA-G (ng/mL) between mother at delivery and cord in GDM.

We verified the possible correlation between the neonatal and maternal amounts of sHLA-G at delivery in both GDM and physiological pregnancies. We observed a statistically significant positive correlation in physiological pregnancies (Ctr) (*r* = 0.20, *p* = 0.03) (Figure 5(a)), while no significant correlation was observed in GDM pregnancies (*r* = 0.08, *p* = 0.76) (Figure 5(b)).

At delivery, in physiological pregnancies, the sHLA-G levels were almost twice in mothers than in the corresponding baby while, in GDM, the sHLA-G levels were greater in babies than in the corresponding mother. The ratio between the sHLA-G median value of the mothers and that of their babies was 1.78 in controls and 0.85 in GDM.

## 4. Discussion

Gestational diabetes mellitus (GDM) is a pregnancy-related complication caused by both genetic predisposition and environmental triggers [1]. We previously reported a direct correlation between low levels of pregnancy-associated plasma protein A (PAPP-A) and GDM onset in a case-control study of first trimester pregnant women [11], and in a subsequent study, we identified the high soluble plasma HLA-G levels as inflammation markers in pregnancies complicated by GDM [12].

Here, we considered mother/newborn couples in healthy and GDM pregnancies. Besides protein levels, we studied the role of HLA-G 14 bp insertion/deletion (3′UTR) and PAPP-A A/C (rs7020782) polymorphisms as possible genetic markers predisposing to GDM, and we further investigated the genetic epistasis between these two genes on GDM risk.

Analyzing the distribution of HLA-G and PAPP-A genotypes in GDM and healthy mothers and the corresponding children, we found that HLA-G 14 bp del/del genotype was slightly more frequent in GDM mothers (OR = 1.7) and babies born to GDM mothers (OR = 2.1) than in controls (healthy mothers and babies born to healthy mothers). PAPP-A C/C genotype was more frequent in GDM mothers (OR = 2.5) and in babies born to GDM mothers (OR = 1.8) than in controls (Table 2), although not reaching statistical significance. Our results are in line with previous observations in pregnancy disorders by Suzuki and colleagues, who firstly reported a correlation between the PAPP-A polymorphism at exon 14 and recurrent pregnancy loss in a large case-control study comprising women with two or more pregnancy losses. The authors found that women carrying the PAPP-A C allele had a higher risk of miscarriage [20].

The epistatic interaction between HLA-G and PAPP-A genotypes revealed a higher frequency of carriers of HLA-G del/del and PAPP-A C/C genotypes in GDM mothers (OR = 9.5) and their babies (OR = 2.8) compared to controls. However, the absence of a statistical significance induces to question on the predictive value of these polymorphisms in GDM onset (Table 3).

To gain insight, we used the correspondence analysis which relates genetic with nongenetic qualitative tracts and gives a picture of factors that most of all mark and distinguish healthy from GDM pregnant women and babies. The nearer the variables are to a specific subgroup in the plot, the stronger their influence on it. Among covariates, HLA-G del/del and PAPP-A C/C genotypes distinguished children born to GDM mothers from those born to healthy ones (Figure 1()), suggesting a possible major paternal signature. On the contrary, clinical variables were nearer to GDM mothers, and no correlation with genetic background was observed, thus excluding a genetic risk linked to the maternal HLA-G 14 bp and PAPP-A A/C genotypes (Figure 1()).

Recently, Svendsen and colleagues confirmed that the HLA-G 14 bp deletion allele shows higher soluble HLA-G1 (sHLA-G) levels than the 14 bp insertion allele [23]. In our study, mothers affected by GDM showed significantly increased sHLA-G levels compared to healthy mothers and those carrying the HLA-G 14 bp del/del genotype produced more sHLA-G at the second trimester and at delivery than healthy mothers (Figure 2). Interestingly, we observed a genetic pressure exerted by the fetal genotype on the maternal sHLA-G production and a peak was reached in diabetic mothers with a heterozygous HLA-G 14 bp del/ins baby and in controls with homozygous HLA-G 14 bp del/del baby (Figure 2). Fetal influence on maternal HLA-G production has been recently proposed by Dahl and coworkers in normal pregnancies [24], and here, we confirm this observation in GDM pregnancies, thus suggesting a possible contribution of the paternal-inherited allele in the cross-control of inflammation.

The cord blood of babies born to GDM mothers showed higher sHLA-G concentrations compared to that of those born to healthy mothers. In particular, those born to GDM mothers and carrying the HLA-G 14 bp del/del genotype showed the highest sHLA-G levels. Similar results were reported in babies of rheumatic pregnancies [25], reinforcing the hypothesis that high amounts of sHLA-G in the cord might represent the immunosignature of a maternal inflammatory milieu (Figure 3).

We verified also the correlation between the PAPP-A A/C genotypes and the protein amounts in GDM and healthy mothers during pregnancy. In the second trimester, we observed an opposite trend in PAPP-A production with respect to the PAPP-A genotypes comparing GDM and healthy pregnancies (Figure 4). PAPP-A A/C heterozygous GDM mothers showed a significant increase of PAPP-A levels during the trimesters with respect to the other genotypes and the controls (Figure 4). Interestingly, in a recent study on 210 samples of follicular fluid from antral follicles donated by 50 volunteer women, the authors showed a significant correlation between the PAPP-A A/C polymorphism (rs7020782) and the activity and levels of PAPP-A proteins in human follicles [26]. Women carrying the C/C genotype showed statistically significantly lower activity and levels of PAPP-A in follicular fluid compared to women carrying the A allele. The C/C carriers also displayed reduced levels of estradiol and increased levels of antimullerian hormone and androgen. The authors also observed an influence of the PAPP-A A/C genotype on the follicular fluid levels of bioactive insulin-like growth factor, thus suggesting a possible underlying aberrant control of insulin-like growth factor activity [26].

Finally, we correlated the amounts of neonatal and maternal sHLA-G at delivery in GDM and healthy pregnancies. We observed a significant positive correlation only in physiological pregnancies (*p* = 0.03, Figure 5). It has been recently proposed that a substantial proportion of maternal sHLA-G amounts derives from the placenta, rather than being produced by maternal cells alone. Hackmon et al. found that cord blood sHLA-G concentration was one-fifth of that in maternal blood, and Klitkou et al. observed that cord blood sHLA-G levels were one-third of those in maternal blood [9, 27]. Taken together, these data suggest that at delivery, in physiological pregnancies, the cord sHLA-G levels are lower than those in maternal blood. That has been confirmed in our study. In fact, in our controls, the median levels of fetal sHLA-G were nearly one-half of those in the corresponding mothers. However, in GDM, the proportion was inverted because of the overproduction of sHLA-G by newborns. The ratio between the median sHLA-G concentrations in mothers at delivery and neonates was 1.78 in controls, while 0.85 in GDM. Thus, the great amounts of sHLA-G in the GDM cord blood might be conditioned by both fetal HLA-G 14 bp del/del genotype and maternal disease.

## 5. Conclusions

We first observed an involvement of HLA-G and PAPP-A gene-protein interaction in GDM susceptibility; thus, we highlight a possible contribution of the fetus in balancing maternal inflammation.

## Figures and Tables

**Table 1:** Major clinical characteristics of gestational diabetes mellitus (GDM) mothers and healthy mothers (controls). Continuous variables are expressed as mean (sd), categorical variables as *n* (%).

Clinical data	GDM *n* = 26	Controls *n* = 151	*p* value
Age	35.3 (5.4)	34.3 (4.6)	0.35
First trimester week maternal body mass index	24.8 (4.8)	22.6 (3.6)	0.01
Maternal weight at first trimester of gestation (kg)	66.0 (14.0)	61.3 (11.0)	0.05
Maternal weight at delivery (kg)	74.6 (14.5)	72.9 (11.4)	0.52
Maternal BMI ≥ 25	12 (46%)	31 (21%)	0.005
Week of gestation at delivery	38.2 (2.5)	39.1 (1.5)	0.01
Birth weight (g)	3261 (562)	3243 (461)	0.86
Birth weight > 90 centile	6 (27%)	11 (8%)	0.01
Cesarean section	10 (38%)	57 (38%)	0.96
Newborn gender (males)	10 (38%)	83 (55%)	0.15
First gestation	13 (50%)	66 (44%)	0.35
Induction	9 (35%)	40 (26%)	0.48
Familiarity for diabetes	14 (54%)	39 (26%)	0.009

**Table 2:** Distribution of the HLA-G 14 bp insertion/deletion (ins/del) and PAPP-A A/C alleles and genotypes among GDM mothers and children versus controls. Absolute and percentage frequencies are reported. OR: odds ratio; 95% CI: 95% confidence interval.

		GDM mothers (*N* = 24)		Healthy mothers (*N* = 151)		OR (95% CI)	*p*
*N*	%	*N*	%		

HLA-G 14 bp ins/del genotypes	del/del	7	29	40	26	1.7 (0.4–2.5)	0.98
	del/ins	13	54	75	50	1.0 (0.4–2.7)	0.50
	ins/ins	4	17	36	24	Reference	
HLA-G 14 bp alleles	DEL	27	56	155	51	1.2 (0.7–2.2)	0.53
	INS	21	44	147	49	Reference	

		Children born to GDM mothers (*N* = 20)		Children born to healthy mothers (*N* = 135)		OR (95% CI)	*p*
*N*	%	*N*	%		

HLA-G 14 bp ins/del genotypes	del/del	8	40	31	23	2.1 (0.6–7.6)	0.27
	del/ins	8	40	72	53	0.9 (0.3–3.2)	0.86
	ins/ins	4	20	32	24	Reference	
HLA-G 14 bp alleles	DEL	24	60	134	50	1.5 (0.8–3.0)	0.22
	INS	16	40	136	50	Reference	

		GDM mothers (*N* = 24)		Healthy mothers (*N* = 151)		OR (95% CI)	*p*
*N*	%	*N*	%		

PAPP-A genotypes	AA	8	33	74	49	Reference	
	AC	13	54	66	44	1.8 (0.7–4.7)	0.21
	CC	3	13	11	7	2.5 (0.6–11)	0.22
PAPP-A alleles	A	29	60	214	71	Reference	
	C	19	40	88	29	1.6 (0.8–3.0)	0.14

		Children born to GDM mothers (*N* = 20)		Children born to healthy mothers (*N* = 135)		OR (95% CI)	*p*
*N*	%	*N*	%		

PAPP-A genotypes	AA	10	50	66	49	Reference	
	AC	7	35	58	43	0.8 (0.3–2.2)	0.66
	CC	3	15	11	9	1.8 (0.4–7.6)	0.42
PAPP-A alleles	A	27	67	190	70	Reference	
	C	13	33	80	30	1.1 (0.6–2.3)	0.71

**Table 3:** Distribution of the HLA-G 14 bp insertion/deletion (ins/del) and PAPP-A A/C genotypic combinations among GDM mothers and children versus the controls. OR: odds ratio; 95% CI: 95% confidence interval.

Genotype combinations	PAPP-A A/C	GDM mothers (*N* = 24)		Healthy mothers (*N* = 151)		OR (95% CI)	*p*
		*N*	%	*N*	%		

HLA-G 14 bp ins/del							
del/del	AA	2	8	19	13	Reference	
	AC	3	12	19	13	1.5 (0.2–10.0)	0.62
	CC	2	8	2	1.3	9.5 (0.8–109)	0.07
del/ins	AA	6	25	37	24	1.54 (0.3–8.4)	0.50
	AC	7	29	33	22	2.0 (0.4–10.7)	0.68
	CC	0	0	5	3	Undetermined	
ins/ins	AA	0	0	18	12	Undetermined	
	AC	3	12	14	9	2.0 (0.3–13.8)	0.47
	CC	1	4	4	3	2.4 (0.2–33.0)	0.52

Genotype combinations	PAPP-A A/C	Children born to GDM mothers (*N* = 20)		Children born to healthy mothers (*N* = 135)		OR (95% CI)	*p*
		*N*	%	*N*	%		

HLA-G 14 bp ins/del							
del/del	AA	2	10	11	8	Reference	
	AC	4	20	16	12	1.4 (0.2–8.8)	0.74
	CC	2	10	4	3	2.8 (0.3–26.7)	0.38
del/ins	AA	7	35	40	30	1.0 (0.2–5.3)	0.96
	AC	1	5	29	21	0.2 (0–2.3)	0.19
	CC	0	0	3	2	Undetermined	
ins/ins	AA	1	5	15	11	0.4 (0–4.6)	0.44
	AC	2	10	13	10	0.8 (0.1–7.0)	0.88
	CC	1	5	4	3	1.4 (0.1–19.6)	0.81
